# Promotion of early and exclusive breastfeeding in neonatal care units in rural Rwanda: a pre- and post-intervention study

**DOI:** 10.1186/s13006-022-00458-9

**Published:** 2022-02-22

**Authors:** Saidath Gato, Francois Biziyaremye, Catherine M. Kirk, Chiquita Palha De Sousa, Alain Mukuralinda, Hamissy Habineza, Maya Asir, Himali de Silva, Marie Louise Manirakiza, Egide Karangwa, Alphonse Nshimyiryo, Alex Tugume, Kathryn Beck

**Affiliations:** 1 Partners In Health/Inshuti Mu Buzima, Rwinkwavu, Rwanda; 2 Boston Children’s Hospital, Boston, USA; 3 MAITS, London, UK; 4 Rwinkwavu District Hospital, Ministry of Health, Kigali, Rwanda

**Keywords:** Neonatal nutrition, Small and sick newborns, Rwanda,, Exclusive breastfeeding**,**, Nurturing care, Baby-friendly

## Abstract

**Background:**

Early initiation of breastfeeding after birth and exclusive breastfeeding for the first six months improves child survival, nutrition and health outcomes. However, only 42% of newborns worldwide are breastfed within the first hour of life. Small and sick newborns are at greater risk of not receiving breastmilk and often require additional support for feeding. This study compares breastfeeding practices in Rwandan neonatal care units (NCUs) before and after the implementation of a package of interventions aimed to improve breastfeeding.

**Methods:**

This pre-post intervention study was conducted at two district hospital NCUs in rural Rwanda from October–December 2017 (pre-intervention) and September 2018–March 2019 (post-intervention). Only newborns admitted before their second day of life (DOL) were included. Data were extracted from patient charts for clinical and demographic characteristics, feeding, and patient outcomes. Exclusive breastfeeding at discharge was based on last recorded infant feeding on the day of discharge. Logistic regression analysis was used to evaluate factors associated with exclusive breastfeeding at discharge.

**Results:**

Pre-intervention, 255 newborns were admitted in the NCUs and 793 were admitted in post-intervention. Exclusive breastfeeding on the day of birth (DOL0) increased from 5.4% (12/255) to 35.9% (249/793). At discharge, exclusive breastfeeding increased from 69.6% (149/214) to 87.0% (618/710). The mortality rate decreased from 16.1% (41/255) to 10.5% (83/793). Factors associated with greater odds of exclusive breastfeeding at discharge included admission during the post-intervention period (aOR 4.91; 95% CI 1.99, 12.11), and admission for infection (aOR 2.99; 95% CI 1.13, 7.93). Home deliveries (aOR 0.15; 95% CI 0.05, 0.47), preterm delivery (aOR 0.36; 95% CI 0.15, 0.87) and delayed first breastmilk feed (aOR 0.04 for DOL3 vs. DOL0; 95% CI 0.01, 0.35) reduced odds of exclusive breastfeeding at discharge.

**Conclusions:**

Expansion and adoption of evidenced-based guidelines, using innovative approaches, aimed at the unique needs of small and sick newborns may help to improve earlier initiation of breastfeeding, decrease mortality, and improve exclusive breastfeeding on discharge from hospital among small and sick newborns. These interventions should be replicated in similar settings to determine their effectiveness.

## Background

Breastfeeding plays a paramount role in child survival, development and maternal health [1]. Early initiation of breastfeeding within one hour after birth and exclusive breastfeeding for the first six months is recommended by the World Health Organization (WHO) [2]. Immediate and early initiation of breastfeeding after birth is associated with better neonatal outcomes, in low- and middle-income countries (LMICs), such as reduction of neonatal deaths, length of hospital stay for sick infants and neonatal infections [3, 4]. A meta-analysis of LMIC settings, showed that non-breastfed infants under six months of age have a 14 times higher risk of death, and partially breastfed infants have a 4.8-fold higher risk of death compared to predominantly breastfed infants [5].

Even though the majority of deliveries globally are attended by skilled healthcare providers, only 42% of newborns are breastfed within the first hour of life worldwide [6]. Small and sick newborns requiring inpatient care after birth face unique challenges since the neonatal care unit environment is not always conducive for initiation of early and exclusive breastfeeding, including in settings such as Denmark, when an infant requires mechanical ventilation or is separated from the mother [7]. In addition, small and sick newborns may experience difficulties breastfeeding, or are too immature or unstable to breastfeed immediately after birth, so mothers require specialized support to establish and maintain their milk supply [8, 9]. Despite the strong recommendation from WHO that maternity and newborn services have trained and competent staff who can provide successful breastfeeding support to lactating mothers [10], they have noted that early breastfeeding is compromised by inappropriate procedures, such as infant-mother separation, performed by healthcare providers and outdated policies [6].

Many interventions exist at community and facility levels worldwide to promote early and exclusive breastfeeding. UNICEF and WHO launched the Baby-Friendly Hospital Initiative (BFHI) in 2009 to integrate breastfeeding with maternal and newborn care in hospitals. Through this multi-level approach, hospitals must implement the “Ten Steps to Successful Breastfeeding” to attain BFHI status, including having a breastfeeding policy, allowing rooming-in, and providing adequate training to staff [10, 11]. Specific recommendations for expanding BFHI to include the unique needs of small and sick newborns have also been developed [9, 12]. Additionally, utilizing lactation consultants for breastfeeding support and lactation education has shown to increase rates of initiation, duration of any breastfeeding and exclusive breastfeeding compared to usual practices in a systematic review of high-income countries [13]. Peer counseling approaches have also demonstrated improved early and continuous exclusive breastfeeding in an urban United States hospital setting [14].

In Rwanda, 81% of newborns are breastfed within one hour of life and 87% are exclusively breastfed up to six months [15], however little is known about rates among small and sick newborns. Data from a neonatal care unit (NCU) in rural Rwanda found a large proportion of small and sick newborns had breastfeeding difficulties after discharge leading to use of infant formula and poor growth [16]. To address this, Partners In Health/Inshuti Mu Buzima (PIH/IMB) in collaboration with the Ministry of Health, implemented interventions for breastfeeding support for newborns and infants with feeding difficulties in NCUs. This included training of healthcare providers, breastfeeding counselling for mothers and health system strengthening to promote early and exclusive breastfeeding. This study aims to compare breastfeeding rates of newborns admitted to district hospital neonatal special care units before and after the implementation of a package of interventions aimed to improve breastfeeding practices.

## Methods

### Study setting

We conducted this study in the Rwinkwavu District Hospital (RDH) and Kirehe District Hospital (KDH) NCUs. RDH and KDH are Rwandan Ministry of Health public hospitals located in Kayonza and Kirehe Districts in the eastern province of Rwanda. Both RDH and KDH have been supported by PIH/IMB, an international non-governmental organization, since 2005 and 2007, respectively. RDH supervises eight health centers in its catchment area with a population of 215,555 and KDH supervises 16 health centers in its catchment area with a population of 384,776 [17], in addition to two health centers and over 60,000 people in a refugee camp in the catchment area [18]. The NCUs provide care for small and sick newborns and are equipped with incubators, radiant warmers, syringe pumps, phototherapy, oxygen and continuous positive airway pressure machines for the management of common neonatal conditions. They are staffed by nurses, with an average nurse to patient ratio of 1 to 8.5 [19], and general practitioners, with mentorship by a pediatrician and midwife. Typically, there is one general practitioner who conducts rounds on patients on the neonatal unit each day. The education level of the nurses vary, and include, either a general nurse with a two-year diploma (A1 level) or a general nurse with a Bachelor’s degree (A0 level).

### Intervention

Several inputs were introduced into the hospital neonatal care units to promote exclusive breastfeeding, including porridge for mothers and water filters to provide a high calorie, high protein supplement and ensure adequate hydration; pillows for more comfortable breastfeeding positioning; screens for mothers to breastfeed or express breastmilk privately; refrigerator and materials for storage of expressed breastmilk, and educational posters promoting exclusive breastfeeding and Kangaroo Mother Care (KMC). In addition to these inputs, a training was conducted in February 2018 called Working with Infants with Feeding Difficulties delivered by two Speech and Language Therapists who are experts in infant feeding. A description of that training package and a case study from its implementation in Rwanda has been described elsewhere [20]. As a result of the training and in an effort to ensure sustainability of the skills learned during the training, each hospital hired two Expert Mothers to serve as peer counsellors to support mothers in assessing breastfeeding readiness, improve positioning and attachment, and create a breastfeeding-friendly, caring environment for mothers with a focus on one-to-one as well as group counselling. The Expert Mothers were chosen based on criteria including previously having an infant in the neonatal unit and commitment to sharing her experience with other mothers. The Expert Mothers are trained on the Working with Infants with Feeding Difficulties package, provided with a job aid and tablet loaded with Global Health Media videos for counselling of mothers.

### Study design and population

We conducted a pre-post study. We included all newborns admitted to the RDH and KDH neonatology units in two periods including pre-intervention from October 2017 to December 2017 and post-intervention from September 2018 to March 2019 who were admitted before their second day of life. The manuscript was prepared following the Standards for Quality Improvement Reporting Excellence (SQUIRE 2.0) Guidelines [21].

### Data collection

Data from nationally standardized neonatology patient charts is routinely collected by trained data collectors on a structured paper, two-page form and then entered in a Microsoft Access database. Data completeness and accuracy were checked through routine data quality assessment activities that are conducted by monitoring and evaluation staff. Types of data collected about the infant include the infant’s reason for admission, day of life on admission, relevant perinatal history, length of stay, and discharge outcomes. Data collected about the mother includes maternal history, such as age, gravida, and para, and type of delivery. A very detailed feeding and weight gain history is recorded on the paper form, including the nutrition method through which the infant was receiving nutritional support (via the breast, via a naso-gastric tube, via a cup, or nil per os [NPO, nothing by mouth].) and the nutrition type (breastmilk only, breastmilk and artificial milk, artificial milk only, or intravenous fluids).

### Definition of variables

Our primary outcome was exclusive intake of breast milk at the time of discharge among infants discharged alive. Data on the infant’s feeding history which was last recorded in the patient chart on the day of discharge was used to assess exclusive breastfeeding at the time of discharge. Day of life 0 (DOL0) referred to the child’s day of birth. Newborns exclusively fed breastmilk were defined as the feeding type recorded in the patient’s chart as ‘only breast milk’, regardless of the method of feeding (i.e., via breast, via naso-gastric tube, etc.). Fed on breast was defined as the method of feeding recorded in the patient’s chart as ‘only on the mother’s breast’ (i.e., not via cup, not via naso-gastric tube, etc.). Low birthweight (LBW) was defined as any birth below 2,500 g and premature births are births before 37 weeks gestation. Home delivery was defined as a birth that takes place in the community outside of the care of a skilled healthcare provider, regardless of whether the home delivery was planned delivery or a precipitous delivery.

### Data analysis

We described sociodemographic characteristics of infants and their mothers, and clinical and feeding characteristics of infants using frequencies and percentages for categorical data and median and interquartile ranges for continuous data. We conducted bivariate analysis using Chi-square test to compare the pre- and post-intervention periods for all categorical sociodemographic, clinical and feeding characteristics described for infants with data recorded unless a cell contained a value of less than five, in which case Fisher’s exact test was used. Wilcoxon Ranksum test was used for bivariate analysis of continuous variables for infants with data recorded. We assessed change in mortality from pre- to post-intervention using multivariable logistic regression controlling for the child’s condition, birthweight in grams, and child’s sex. Then, we used multivariable logistic regression models to identify predictors associated with the outcome ‘exclusive breastfeeding on discharge’, built using backward stepwise procedures for all variables significant at α = 0.20 in bivariate analyses. All factors significant at the α = 0.05 significance level were retained in the final model. The data were analyzed using Stata v.15.1 (Stata Corp, College Station, TX, USA).

### Ethics

The study received ethical approval from the Rwanda National Ethics Committee (No. 105/RNEC/20). Data was captured through review of routine records and so additional informed consent specific to this study was not required.

## Results

In total, 255 newborns were admitted in the neonatology care units during the pre-intervention and 793 were admitted in the post-intervention periods (Table 1). There were no significant differences in admissions for prematurity at pre-intervention compared to post-intervention (40.0% [*n* = 96/240] vs. 40.6% [*n* = 309/762], *p* = 0.88), maternal age (50.0% [*n* = 12/240] age 25–34 years vs. 43.7% [*n* = 332/759], *p* = 0.24) maternal gravidity (27.1% [*n* = 66/244] primigravida vs 31.5% [*n* = 238/755], *p* = 0.40), and age on admission (83.1% [*n* = 212/255] admitted on DOL0 vs. 85.4% [*n* = 677/793, *p* = 0.39). Compared to the pre-intervention period, infants were also more likely than in the post-intervention period to be delivered by caesarean section (31.5% [*n* = 79/251] vs. 41.1% [*n* = 315/767], *p* = 0.01) and weigh more than 1,500 grams (86.6% [*n* = 220/254] vs. 91.9% [*n* = 722/786], *p* = 0.02).

**Table 1:** Demographic and clinical characteristics of newborns admitted to Rwinkwavu and Kirehe District Hospital neonatal care units from October – December 2017 (pre-intervention) and September 2018 – March 2019 (post-intervention)

Variable	Pre-intervention (*N* = 255)		Post-intervention (*N* = 793)		Bivariate analysis*		
	*n*	%	*n*	%	*p*-value	Degrees of freedom	Test statistic
Hospital of admission					0.003	1	8.98
Kirehe	141	55.3	521	65.8			
Rwinkwavu	114	44.7	272	34.3			
Place of child's birth	(*n* = 253)		(*n* = 779)		0.02	3	9.56
Hospital	165	65.2	531	68.2			
Health Center	70	27.7	158	20.3			
Home	16	6.3	69	8.9			
Other	2	0.8	21	2.7			
Type of delivery	(*n* = 251)		(*n* = 767)		0.01	1	7.34
Vaginal	172	68.5	452	58.9			
Caesarean section	79	31.5	315	41.1			
Sex of child	(*n* = 210)		(*n* = 781)		< 0.001	1	15.73
Male	83	39.5	429	54.9			
Female	127	60.5	352	45.1			
Birthweight category	(*n* = 254)		(*n* = 786)		0.02	3	9.89
Extremely low birthweight, < 1000 g	13	5.1	16	2.0			
Very low birthweight, < 1500 g	21	8.3	48	6.1			
Low birthweight, < 2500 g	94	37.0	342	43.5			
Normal birthweight, ≥ 2500 g	126	49.6	380	48.4			
Prematurity category recorded in hospital register	(*n* = 240)		(*n* = 762)		0.88	1	0.02
Term	144	60.0	453	59.5			
Preterm	96	40.0	309	40.6			
Gestational age category (if gestational age in weeks reported)	(*n* = 225)		(*n* = 683)		0.05	3	8.02
Extremely preterm < 28 weeks	9	4.0	12	1.8			
Very preterm 28–31 weeks	21	9.3	39	5.7			
Moderate/late 32–36 weeks	51	22.7	179	26.2			
Term 37 + weeks	144	64.0	453	66.3			
Mother's Age	(*n* = 240)		(*n* = 759)		0.24	3	4.20
< 20 years	19	7.9	79	10.4			
20–24 years	52	21.7	198	26.1			
25–34 years	120	50.0	332	43.7			
35 + years	49	20.4	150	19.8			
Gravidity (# pregnancies) of mother	(*n* = 244)		(*n* = 755)		0.40	2	1.85
1 pregnancy	66	27.1	238	31.5			
2–4 pregnancies	121	49.6	358	47.4			
5 + pregnancies (grand multigravida)	57	23.4	159	21.1			
Parity (# viable deliveries) of mother	(*n* = 244)		(*n* = 757)		0.01	2	9.73
1 delivery	79	32.4	330	43.6			
2–4 deliveries	127	52.1	323	42.7			
5 + deliveries (grand multiparous)	38	15.6	104	13.7			
DOL^**a**^ on admission					0.39	1	0.75
DOL 0	212	83.1	677	85.4			
DOL 1	43	16.9	116	14.6			
Diagnosis (not mutually exclusive)							
Preterm, or gestational age < 37 weeks	102	40.0	308	38.8	0.74	1	0.11
LBW^b^, or birthweight < 2500 g	123	48.2	406	51.2	0.41	1	0.68
Infection	141	55.3	328	41.4	< 0.001	1	15.15
HIE^c^/asphyxia	29	11.4	70	8.8	0.23	1	1.46
Other	23	9.0	145	18.3	< 0.001	1	12.31

^a^Day of Life

^b^Low Birthweight

^c^Hypoxic Ischemic Encephalopathy

The percentage of infants who were fed on the breast on DOL0 increased from 5.8% (*n* = 13/223) to 35.6% (*n* = 247/694), (*p* < 0.001) and exclusively breastfed on DOL0 increased from 5.4% (*n* = 12/255) to 35.9% (*n* = 249/793) (*p* < 0.001) (Table 2). On DOL1, feeding on the breast increased from 30.5% (*n* = 75/246) to 68.6% (*n* = 532/776) (*p* < 0.001) and exclusive feeding on breastmilk increased from 37.4% (*n* = 92/255) to 70.2% (*n* = 545/793) (*p* < 0.001). For newborns discharged alive, the proportion fed on the breast increased from 59.8% (*n* = 128/255) to 84.7% (*n* = 601/793) (*p* < 0.001) and the proportion exclusively feeding on breastmilk increased from 69.6% (*n* = 149/255) to 87.0% (*n* = 618/793) (*p* < 0.001). Introduction of first breast milk feeding on DOL0 increased from 7.1% (*n* = 18/255) pre-intervention to 31.9% (*n* = 253/793) in post-intervention (*p* < 0.001).

**Table 2:** Breastfeeding timing and breastfeeding discharge status of newborns admitted to Rwinkwavu and Kirehe District Hospital neonatal care units between a pre- and post- breastfeeding intervention

Variable	Pre-intervention (*n* = 255)		Post-intervention (*n* = 793)		Bivariate analysis		
	*n*	%	*n*	%	*p*-value*	Degrees of freedom	Test statistic
Newborn fed on breast day of life (DOL^a^) 0	(*n* = 223)		(*n* = 694)		< 0.001	1	61.64
Yes	13	5.8	247	35.6			
No	158	70.9	381	54.9			
Not documented	52	23.3	66	9.5			
Newborn exclusively fed breastmilk on DOL 0					< 0.001	1	65.07
Yes	12	5.4	249	35.9			
No	159	71.3	379	54.6			
Not documented	52	23.3	66	9.5			
Newborn fed on breast on DOL 1	(*n* = 246)		(*n* = 776)		< 0.001	1	118.63
Yes	75	30.5	532	68.6			
No	156	63.4	205	26.4			
Not documented	15	6.1	39	5.0			
Newborn exclusively fed breastmilk on DOL 1					< 0.001	1	89.32
Yes	92	37.4	545	70.2			
No	137	55.7	191	24.6			
Not documented	17	6.9	40	5.2			
Newborn fed on breast on DOL 2	(*n* = 238)		(*n* = 736)		< 0.001	1	91.11
Yes	107	45.0	568	77.2			
No	114	47.9	133	18.1			
Not documented	17	7.1	35	4.8			
Newborn exclusively fed breastmilk on DOL 2					< 0.001	1	64.65
Yes	135	56.7	600	81.5			
No	87	36.6	100	13.6			
Not documented	16	6.7	36	4.9			
Newborn fed on breast DOL 7	(*n* = 195)		(*n* = 441)		< 0.001	1	33.97
Yes	106	54.4	331	75.1			
No	68	34.9	66	15.0			
Not documented	21	10.8	44	10.0			
Newborn exclusively fed breastmilk on DOL 7					< 0.001	1	25.62
Yes	124	63.6	351	79.6			
No	48	24.6	43	9.8			
Not documented	23	11.8	47	10.7			
Newborn fed on breast on discharge day if discharged alive					< 0.001	1	50.81
Yes	128	59.8	601	84.7			
No	41	19.2	38	5.4			
Not documented	45	21.0	71	10.0			
Newborn fed breastmilk on discharge day if discharged alive					< 0.001	1	31.42
Yes	149	69.6	618	87.0			
No	19	8.9	12	1.7			
Not documented	46	21.5	80	11.3			
DOL of first documented breastmilk feed					< 0.001	3	91.01
DOL0	18	7.1	253	31.9			
DOL1	99	38.8	330	41.6			
DOL2	66	25.9	105	13.2			
DOL3 or later	72	28.2	105	13.2			

^a^Day of Life

*P-value compares only non-missing data values; "Not documented" is not included in the chi-square test

The median length of hospital stay among infants admitted to the neonatal unit was reduced from eight days in the pre- to seven days in the post-intervention periods (*p* < 0.001) (Tables 3 and 4). The overall mortality rate for all newborns admitted decreased from 16.1% (*n* = 41/255) in pre- to 10.5% (*n* = 83/793) in post-intervention periods (*p* = 0.02). Mortality rate for LBW newborns reduced from the pre-intervention to post-intervention period (23.6% [*n* = 29/255] vs. 15.0% [*n* = 61/793], *p* = 0.03), but remained similar among babies diagnosed with other conditions. Reduced odds of mortality post-intervention were significant when controlling for diagnosis, birthweight, and child’s sex (adjusted odds ratio [aOR] 0.57; 95% confidence interval [CI] 0.34, 0.96).

**Table 3:** Difference in mortality and length of hospital stay among newborns admitted to Rwinkwavu and Kirehe District Hospital neonatal care units before and after breastfeeding interventions

Variable	**Pre-intervention** **(*n* = 255)**		**Post-intervention** **(*n* = 793)**		Bivariate analysis*		
	***n***	**%**	***n***	**%**	***p*-value**	**Degrees of freedom**	**Test statistic**
**LOS**^**a**^**, median [IQR**^**b**^**]**	8	[6, 8]	7	[3, 9]	< 0.001	NA	z = 4.84
**Mortality**							
Yes	41	16.1	83	10.5	0.02	1	5.83
No	214	83.9	710	89.5			
**Mortality by condition**							
Preterm					0.13	1	2.31
Died	26	25.5	57	18.5			
Discharged alive	76	74.5	251	81.5			
LBW^c^					0.03	1	4.89
Died	29	23.6	61	15.0			
Discharged alive	94	76.4	345	85.0			
Hypoxic ischemic encephalopathy (HIE^d^)					0.65	1	0.20
Died	7	24.1	20	28.6			
Discharged alive	22	75.9	50	71.4			
Neonatal infection					0.28	1	1.15
Died	15	10.6	25	7.6			
Discharged alive	126	89.4	303	92.4			
**Mortality by birthweight**							
Extremely low birthweight, < 1000 g					0.12	1	2.43
Died	12	92.3	11	68.8			
Discharged alive	1	7.7	5	31.3			
Very low birthweight, < 1500 g					0.05	1	3.85
Died	6	28.6	26	54.2			
Discharged alive	15	71.4	22	45.8			
LBW, < 2500 g					0.11	1	2.54
Died	11	11.7	23	6.7			
Discharged alive	83	88.3	319	93.3			
Normal birthweight ≥ 2500 g					0.20	1	1.64
Died	11	8.7	21	5.5			
Discharged alive	115	91.3	359	94.5			

^a^Length of Stay

^b^Interquartile Range

^c^Low Birthweight^d^ Hypoxic Ischemic Encephalopathy

^e^Extremely Low Birthweight

^f^Very Low Birthweight

^*^A z value was reported for the Wilcoxon rank sum test, but no degree of freedom

*NA*, not applicable

**Table 4:** Association between intervention and mortality

Study time period	OR^a^	Standard error	z	[95% CI]
Pre-intervention	ref			
Post-intervention	0.57	0.15	-2.10	[0.34, 0.96]

*ref, reference category*

^a^Odds ratio, adjusted for conditions (preterm, HIE, infections), birthweight, and child’s sex

In the final model (Table 5), there was a significant increase in exclusive feeding of breastmilk at discharge if admitted in the post-intervention period (aOR 4.91; 95% CI 1.99, 12.11). Factors associated with increased odds of exclusive breastfeeding at discharge included diagnosis of infection or infection risk (aOR 2.99; 95% CI 1.13, 7.93). Factors associated with reduced odds of exclusive breastfeeding at discharge included home birth (aOR 0.15; 95% CI 0.05, 0.47), prematurity (aOR 0.36; 95% CI 0.15, 0.87), and later timing of first breastmilk feed (DOL2 aOR 0.11; 95% CI 0.01, 0.995; DOL3 or later aOR 0.04; 95% CI 0.005, 0.35).

**Table 5:** Multivariable analysis of demographic and clinical predictors of exclusively feeding on breastmilk at discharge for newborns discharged alive from Rwinkwavu and Kirehe District Hospital neonatal care units from October – December 2017 and September 2018 – March 2019

Variable	Model 1 (Full)				Model 2 (Reduced)			
	Adjusted odds ratio (aOR)	Standard error	z	95% confidence interval (CI)	Adjusted odds ratio (aOR)	Standard error	z	95% confidence interval (CI)
Study time period								
Pre-intervention	ref				ref			
Post-intervention	3.53	2.00	2.24	1.17, 10.72	4.91	2.26	3.46	1.99, 12.11
Hospital of admission								
Kirehe	ref							
Rwinkwavu	0.40	0.21	-1.75	0.14, 1.12				
Place of child's birth								
Hospital	ref				ref			
Health center	0.40	0.30	-1.23	0.09, 1.73	0.44	0.22	-1.68	0.17, 1.15
Home	0.22	0.19	-1.79	0.04, 1.16	0.15	0.09	-3.25	0.05, 0.47
Type of delivery								
Vaginal	ref							
Caesarean-section	0.83	0.64	-0.23	0.19, 3.77				
Sex of child								
Male	ref							
Female	0.63	0.34	-0.84	0.22, 1.83				
Birthweight category								
Extremely low birthweight, < 1000 g	ref							
Very low birthweight, < 1500 g	1.76	3.11	0.32	0.06, 56.25				
LBW^a^, < 2500 g	3.46	5.61	0.76	0.14, 83.08				
Normal birthweight ≥ 2500 g	1.60	2.85	0.26	0.05, 52.78				
Prematurity category recorded in hospital register								
Term	ref				ref			
Preterm	0.31	0.23	-1.57	0.07, 1.34	0.36	0.17	-2.22	0.15, 0.87
Mother's age								
< 20 years	ref							
20–24 years	0.89	1.15	-0.09	0.07, 11.24				
25–34 years	0.45	0.60	-0.60	0.03, 6.06				
35 + years	0.58	0.84	-0.38	0.03, 9.87				
Parity (# viable deliveries) of mother								
1 delivery	ref							
2–4 deliveries	0.76	0.56	-0.38	0.18, 3.21				
5 + deliveries (grand multiparous)	0.59	0.55	-0.56	0.09, 3.71				
Diagnosed with infection or infection risk								
No	ref				ref			
Yes	3.09	1.82	1.91	0.97, 9.83	2.99	1.49	2.21	1.13, 7.93
Diagnosed with HIE^b^/asphyxia								
No	ref							
Yes	0.79	0.75	-0.25	0.12, 5.09				
Day of life (DOL^c^) of first breastmilk feed								
DOL0	ref				ref			
DOL1	0.24	0.26	-1.32	0.03, 2.03	0.24	0.26	-1.32	0.03, 2.00
DOL2	0.12	0.14	-1.81	0.01, 1.19	0.11	0.13	-1.96	0.01, 0.995
DOL3 or later	0.05	0.06	-2.55	0.01, 0.51	0.04	0.04	-2.92	0.005, 0.35

The variance inflation factor (VIF) for collinearity diagnosis among predictor variables in Model 1, ranged between 1.09 and 1.84

*VIF for Model 2, ranged between 1.01 and 1.12*

*ref* reference category

^a^ Low Birthweight

^b^ Hypoxic Ischemic Encephalopathy

^c^ Day of Life

## Discussion

Our study showed that a multi-level intervention, aimed at improving rates of exclusive breastfeeding on a hospital neonatology unit in rural Rwanda, increased early and exclusive breastmilk feeding, and was also associated with a reduced length of stay and decreased mortality among small and sick newborns. These strategies have potential for expansion to other similar contexts, and may improve hospital neonatal outcomes and ensure small and sick newborns will benefit from the well-known long-term benefits of breastfeeding [1].

We found that from the pre- to post-intervention period, significantly more infants were fed on the breast and were exclusively fed breastmilk. We also observed earlier initiation of breastfeeding in the post-intervention and this earlier initiation increased the odds that an infant was discharged exclusively feeding on breastmilk. This is consistent with other literature as it is well known that if milk removal does not occur either by infant suckling or expression by hand or pump, milk secretion will start to decline around day three postpartum [22, 23]. A study from the United States comparing milk expression within one hour after delivery to within 1–6 h after delivery showed that the earlier expression group had earlier lactogenesis stage II (transition from colostrum to copious breast milk production) and resulted in higher milk volume [24]. Another study from the United States showed that milk volume on postpartum day four is predictive of having an adequate milk supply at six weeks [25]. These studies demonstrate the critical need for early expression of breast milk after delivery, whether the infant is able to breastfeed on the breast or if the mother expresses breastmilk and the infant receives breastmilk through other enteral feeding routes (i.e., cup, nasogastric tube).

Mortality among newborns decreased from the pre- to post-intervention period, particularly among infants born with a low birthweight. The association between decreased mortality and exclusive breastfeeding among infants in all settings has been well established in the literature and is often promoted as strong support for initiation of early and exclusive breastfeeding [1, 3–6, 26].

Overall length of hospital stay showed a significant reduction from the pre- to post-intervention period. While hospital neonatology units are meant to be an environment for infants to improve from various illnesses or conditions, a study from a United States hospital has shown that long length of stay in hospitals also increases an infant’s chance of contracting hospital acquired infections [27]. Therefore, the ability to reduce the length of stay for newborns may have an impact on the overall morbidity of the infant. While we did not measure morbidity in this study, early initiation of breastfeeding has been shown to reduce morbidity in newborns in LMIC settings [3], which likely has a positive impact on the total length of hospitalization. Studies from the United States and New Zealand have shown that reducing the time families, particularly mothers, spend in hospitals can also have a significant impact on the mother’s stress, and the family’s economic situation [28, 29]. Reduced length of stay was likely a secondary outcome of improved breastfeeding rates in the post-intervention period. The overall larger number of admissions with a birthweight greater than 1,500 g and gestational age over 32 weeks in the post-intervention period may have also contributed to lower mortality and shorter length of stay.

We found that the location of where the infant is born was associated with whether they are discharged from the hospital with exclusive breastfeeding or not. Infants born at health centers or in the home (including both planned home deliveries and precipitous deliveries) were less likely to be discharged with exclusive breastfeeding, compared to those born in the hospital which has also been seen in other studies of sub-Saharan Africa and LMIC settings [30, 31]. There may be many reasons for this. Infants born at home need to first be transferred to the health center, and subsequently to the hospital which may delay the introduction of breastmilk for those infants, and subsequently impact whether the infant is discharged exclusively breastfeeding. The clinical staff at health centers may also be less experienced in caring for high-risk newborns, and may not follow essential newborn care practices and delay introduction of breastmilk since the infant needs to be clinically stabilized and then transferred to the hospital.

Infants born preterm were less likely to be discharged exclusively breastfeeding compared to infants born at term. Infants born preterm have unique feeding needs that require specialized interventions and management, and a study from Australia has demonstrated similar findings of reduced exclusive breastfeeding rates even among moderate to late preterm newborns compared to term newborns [32]. Similarly, infants admitted for infection risk or neonatal infection in our study had much higher odds of exclusive breastfeeding. These findings are not surprising, as these infants are often term and feed easily. But notably, even when considering all of these factors in multivariate analysis the admission during the post-intervention period was the strongest predictor of exclusive breastfeeding at the time of discharge with nearly double the odds of exclusive breastfeeding compared to the pre-intervention period. Other factors in the neonatal care unit environment may also interfere with early and exclusive breastfeeding, including delayed initiation of KMC, or skin-to-skin contact, especially for sick newborns as seen in high- and middle-income country setting [33]. We were unable to measure timing or duration of KMC but this is an area that warrants further attention to reduce breastfeeding barriers.

Our study has some limitations. First, we used routinely collected data for the study, which results in some missing data and reliance on clinician skills in completion of medical files. In addition, precise measurement of gestational age is a challenge in Rwanda like in other low- and middle-income countries where availability of ultrasound dating is limited. Due to the use of routine data, it was not possible to reliably discriminate between newborns with infection or those with risk of infection and so we included all of these newborns in our sample. We also had a small sample size of patients born with HIE and those born with extremely low birthweight, which prevented measurement of the impact of interventions on these subsets. In addition, our pre-post design without a comparison group in only two hospitals does not allow us to determine effectiveness of the intervention as changes over time could be influenced by other factors and may not be generalizable to other hospitals. Another potential limitation of this study is that the two study periods, pre-intervention from October 2017 to December 2017 and post-intervention from September 2018 to March 2019, were not exactly aligned which may have led to differences in characteristics among the study populations, including the nutritional status of the mother in both the prenatal and postpartum periods. We also had a small proportion of cases of home births (6.3% in the pre-intervention and 8.9% in the post-intervention group). While most deliveries in Rwanda take place in a health facility (greater than 90%) [15], we do not know whether the home births in our study were planned deliveries or precipitous deliveries, which may also have implications for the prenatal care that was received, as well as other factors.

## Conclusions

A multi-level breastfeeding intervention was feasible to implement in rural Rwandan hospitals and was associated with improvements in earlier initiation of breastmilk feeding, exclusive breastfeeding on discharge, reduced length of stay, and decreased mortality among infants admitted to the hospital neonatal care units. Adoption of evidenced-based guidelines such as the Baby Friendly Hospital Initiative aimed at the unique needs of small and sick newborns and innovative interventions should be expanded and adapted in similar settings to improve outcomes for these infants.

## Data Availability

The datasets used and/or analyzed during the current study are available from the corresponding author on reasonable request.

## Abbreviations

BFHI: Baby-Friendly Hospital Initiative
DOL: Day of life
HIE: Hypoxic ischemic encephalopathy
LBW: Low birth weight
LMIC: Low- and middle-income countries
KMC: Kangaroo Mother Care
KDH: Kirehe District Hospital
NCU: Neonatal Care Unit
PIH/IMB: Partners In Health/Inshuti Mu Buzima
RDH: Rwinkwavu District Hospital
